# Validation of the Chinese Version of Relaxation Sensitivity Index: A Tool for Predicting Treatment Effect in Mindfulness Interventions

**DOI:** 10.3389/fpubh.2021.809572

**Published:** 2021-12-20

**Authors:** Jieting Zhang, Christina M. Luberto, Qi Huang, Jin Kuang, Juan Zhong, Albert Yeung, Liye Zou

**Affiliations:** ^1^Department of Psychology, Shenzhen University, Shenzhen, China; ^2^Institution for Mental Health, Shenzhen University, Shenzhen, China; ^3^Massachusetts General Hospital and Harvard Medical School, Boston, MA, United States; ^4^Springfield College, Springfield, MA, United States; ^5^Depression Clinical and Research Program, Department of Psychiatry, Massachusetts General Hospital and Harvard Medical School, Boston, MA, United States; ^6^Exercise Psychophysiology Laboratory, School of Psychology, Shenzhen University, Shenzhen, China

**Keywords:** relaxation sensitivity, relaxation-induced anxiety, anxiety sensitivity, factor analysis, validity

## Abstract

**Background:** The Relaxation Sensitivity Index (RSI) measures relaxation-related fears developed and validated in western samples. The RSI captures three facets of fear regarding relaxation: physical, cognitive, and social concerns. This study aimed to translate and identify the factor structure of the Chinese version of the RSI.

**Methods:** In a preliminary study, 26 items were generated mainly by translation and modified from the original RSI. In Study 1, factor analysis and internal consistency reliability analysis were conducted on separated half samples of 597 Chinese college students. In Study 2, test-retest reliability, convergent, and predictive criterion validity were examined based on 465 Chinese college students.

**Results:** Fourteen items were selected based on the factor loading and item prevalence in the preliminary study. Factor analysis based on Study 1 identified three factors: *Social appealing, Social performance*, and *Physical concerns*. In general, the RSI demonstrated good internal consistency (αs = 0.750–0.860), convergent validity and predictive criterion validity, while the test-retest reliability is relatively low (*r*s = 0.525–0.685). Notably, less related to the other two factors, *Social performance concerns* may be a unique factor solely predicting social anxiety (*p* <0.001), but not relaxation-induced anxiety (*p* = 0.442).

**Conclusion:** The Chinese version of the RSI possesses a factor structure different from the western population. The robustness of factor structure and test-retest reliability was not as good as expected. Further research is warranted to explore the validity of the RSI in Chinese samples.

## Introduction

Evidence has been found that mindfulness- and relaxation-based therapies can reduce anxiety levels (1). At the same time, increased anxiety as a result of relaxation training or mindfulness practice has also been reported (2), especially when the attendee feels relaxed (2). This increased anxiety resulting from relaxation has been termed relaxation-induced anxiety (RIA) and is prevalent among chronically anxious and non-anxious individuals in relaxation procedures (3–5).

Under the theoretical framework of anxiety sensitivity which is the fear of arousal-related sensations facing anxiety (6), Luberto and colleagues (7) initially defined the term “relaxation sensitivity” as the fear of relaxation-related sensations involving three dimensions of fears induced by relaxation: fear of physical, cognitive events and negative social consequences (7). Relaxation may make individuals focus more on uncomfortable somatic experiences [e.g., somatic pain, feelings of floating, the release of muscle tension (2, 8)], or have cognitive changes such as perceiving a loss of control or intrusive of worrisome thoughts (8). Negative social consequences may include fears of falling behind or being considered lazy (4, 8–10). Hence, the fear of negative social image or performance is another reason for the negative feeling of relaxation.

Against the background above, Luberto and colleagues (7) developed a 21-item self-reported scale called the Relaxation Sensitivity Index (RSI), investigating its psychometric properties among clinical and non-clinical samples. For both healthy individuals and those with mood and anxiety symptoms, the factor structure of RSI was consistent with the proposed theoretical construct (three factors of physical, cognitive, and social concerns), and the total or sub-scales scores of RSI were positively associated with anxiety and most of its related symptoms (e.g., panic, PTSD and depression). Notably, distinct from each other, relaxation sensitivity and anxiety sensitivity may cause anxiety, but maybe of different kinds. Specifically, relaxation sensitivity rather than anxiety sensitivity may induce RIA (7). Luberto et al. (7) suggested the potential usage of RSI in clinical practice and study. Specifically, it could help identify the attendees with potential adverse outcomes in the relaxation-based therapy or mindfulness-based intervention, as indicated by the RSI scores. Furthermore, interventions (e.g., mindful exercises like yoga or Tai Chi with strong somatic experience versus mindfulness with more cognitive focus) could be tailored to patients' specific relaxation-related concerns (i.e., physical, cognitive, or social concerns). Additionally, RSI provides an assessment tool for examining how the fear of relaxation influences psychological, biological, or behavioral outcomes (e.g., stress, cortisol, and sleep problems).

In this study, we aimed to examine and validate the structure of the Chinese version of RSI and further explore its relationship with anxiety sensitivity and relaxation-induced anxiety. In the preliminary study, we translated and revised the RSI into a Chinese version. In Study 1, the factor structure of the Chinese version RSI was explored among Chinese college students by exploratory factor analysis (EFA) and confirmatory factor analysis (CFA). Internal consistency reliability was also examined. In Study 2, test-retest reliability, convergent validity, and predictive criterion validity of RSI were examined in a subset of participants from Study 1.

## Preliminary Study

The authors (7) of the 21-item RSI scale have granted permission for translation to Chinese. First, the original scale was translated into Chinese by four professors and a graduate student in Psychology with fluent English skills. All items were back-translated into English by a bilingual speaker who was blinded to the original version. Inconsistencies between the back-translated items and the original ones were fully discussed until a consensus was reached. Of note, to be culture-specific and readable, one item (“It scares me when I feel tension release in my muscles.”) was replaced by another one from the initial 39-item tested version by Luberto et al. (“It scares me when my body feels relaxed.”). Notably, East Asian individuals pay more attention to social relationships and tend to compare their social performance with others (11), and the original English RSI contained fewer items on Social concerns than the other factors. To this end, we developed five additional items into the Social concerns involving social relationships and social performance, formulating a 26-item RSI version. The 26 items were finalized based on the feedback received from the author of the original RSI.

An online questionnaire was distributed nationwide, and 313 Chinese college students (30 % males, *M*_age_ = 23.03, *SD* = 6.65) signed in and completed the questionnaire online. All studies were approved by the Human Research Ethics Committee of Shenzhen University (No: PN-2020-43). Informed written consent was obtained from participants before assessments. Participants were informed that they were allowed to withdraw from the study at any time.

Exploratory factor analysis was conducted on the 26 items, by Mplus version 8.3 (12), with categorical indicators, oblique rotation, and weighted least squares estimation with mean and variance adjustment (WLSMV). The best model among the 1~6-factor models was selected according to the scree plot of eigenvalues, model fit, the structure of the factor loadings, and theoretical interpretations. Factor loadings were then examined for the optimal model. We deleted items with loadings lower than 0.40 on all factors or with cross-loadings (i.e., item has substantially high loadings on two or more factors, and their difference of loadings is <0.20) until all item factor loadings were acceptable (13).

The scree plot showed that the curve was clear at the two-factor model, indicating a 1-factor solution (see Supplementary Figure A1). However, the model fit in the 1-factor solution was worse than the 2 or 3-factor solution (see Supplementary Table A1). In the 3-factor structure (see Supplementary Table A2), the first factor with 13 items was about physical concerns (eight items, e.g., “It scares me when my limbs feel heavy.”) and cognitive concerns (five items, e.g., “I do not like to relax because it makes me feel out of control.”). The second factor contained only two items about social appealing concerns (e.g., “I fear that if my body is relaxed, I will not be socially appealing.”). The third factor contained four items about social performance concerns (e.g., “ I do not like to relax because it makes me feel out of contact with others.”). There were six items with cross-loadings and one item with low loading.

Considering the limited number of items related to social appealing concerns, we continued trying the 2-factor solution. In the 2-factor structure, the first factor with 15 items was about physical concerns and cognitive concerns, which was consistent with the first factor in the 3-factor solution; the second factor contained seven items about social concerns, in the perspectives of social appealing and performance as indicated in the second and the third factors of the 3-factor solution. Four items (items 24, 3, 6, and 8) were deleted due to low loading and cross-loadings (Supplementary Table A2). To condense the first factor with 15 items, we deleted some similar items with relatively lower loading, or with less prevalence among the Chinese (e.g., “I do not like activities like meditation because of the way they make my body feel.,” see Supplementary Table A2 for details). This process resulted in a 14-item RSI with a possible 2-factor structure for further psychometric investigation in Study 1 and 2.

## Study 1

The purpose of Study 1 was to explore the factor structure of the 14-item RSI among college students through EFA and to verify the factor model through CFA. It was hypothesized to have (1) the same factor structure of RSI as in the preliminary study and (2) good internal consistency reliability for the total scale and each sub-scale.

### Method

A total of 597 Chinese college students (40.5 % males, *M*_age_ = 20.24, *SD* = 1.431) participated in the study by completing the online questionnaire of the 14-item RSI as developed in the preliminary study. Participants were randomly divided into Sample 1 (*N* = 312, 37.8% males, *M*_age_ = 20.287, *SD* = 1.434) and Sample 2 (*N* = 285, 43.5% males, *M*_age_ = 20.197, *SD* = 1.423) for EFA and CFA respectively. Measurement invariance of RSI was further tested by multi-group CFA (14, 15) across gender, relaxation experience, and age, respectively. We first tested measure invariance subsequently by configural invariance model (M1, equal factor loading patterns but unequal factor loadings and thresholds across groups), metric invariance model (M2, invariant factor loadings across groups), and the scalar invariance model (M3, both the factor loadings and the thresholds equal across groups). We then tested structural invariance by constraining latent means (M4) and latent (co)variance (M5) across groups. Given the well-known limitations of χ2 tests that depend on model complexity and sample size, we used the change in CFI (ΔCFI values <0.010) as evidence for measurement invariance (16). There were no significant differences in age (*t* = 0.765, *p* = 0.444, Cohen's *d* = 0.063) and sex (χ^2^ = 1.999, *df* = 1, *p* = 0.157) between the two samples. Cronbach's alpha was computed for the RSI scale and all sub-scales to assess internal consistency reliability based on Sample 2.

### Results

#### Exploratory Factor Analyses

**Figure 1:**
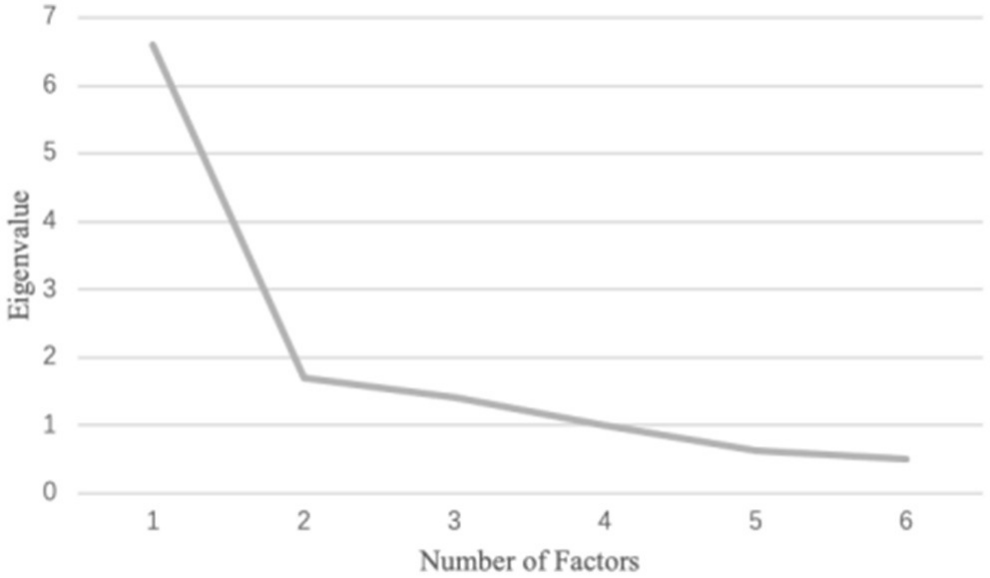
Screen plot for the first exploratory factor analysis with 14 items.

The scree plot (Figure 1) demonstrated a curve at the 2-factor model, and the eigenvalue started to drop below 1 at the 4-factor model. Table 1 suggested the 1-factor model had a relatively low model fit, and the 3-factor model fitted better than the 2-factor model. Though with a better model fit, the 4-factor model extracted two factors related to physical concerns, one of which only contained two items (i.e., “I'm scared of doing relaxing activities because they make me feel vulnerable.” and “When I try to relax my body, I feel like I'm losing control.”). Hence, the 3-factor model was chosen, given its relatively good model fit and practical interpretability. As demonstrated in Table 2, all items had distinctively high loadings in the respective three factors, except that item 8 (i.e., “It scares me when my body feels relaxed.”) cross-loaded in Factor 2 and 3.

**Table 1:** Model fit for EFA (14 items).

	***χ^2^***	***df***	**CFI**	**TLI**	**SRMR**	**RMSEA (90%CI)**
1-factor	734.870	77	0.848	0.821	0.156	0.165 [0.155–0.177]
2-factor	398.026	64	0.923	0.890	0.103	0.129 [0.117–0.142]
3-factor	186.965	52	0.969	0.945	0.061	0.091 [0.077–0.105]
4-factor	77.652	41	0.992	0.981	0.032	0.054 [0.035–0.072]
5-factor	32.612	31	1.000	0.999	0.019	0.013 [0.000–0.045]
6-factor	18.742	22	1.000	1.003	0.014	0.000 [0.000–0.039]

**Table 2:** Factor loadings for the first three-factor model.

**Item**	**Factor**		
	**F1**	**F2**	**F3**
1. 当我让身体放松时, 我担心我会失去外表吸引力 [I worry that when I let my body relax, I will look unattractive.]	**0.947**	−0.006	−0.022
2. 如果我的身体放松下来, 我担心失去社交魅力/吸引力 [I fear that if my body is relaxed, I will not be socially appealing.]	**0.930**	−0.065	0.004
3. 我担心当我让身体放松时, 人们会取笑我 [I worry that when I let my body relax, people will make fun of me.]	**0.629**	0.010	0.201
4. 我不喜欢放松, 因为这让我感到与他人失去了联系 [I do not like to relax because it makes me feel out of contact with others.]	**0.500**	0.117	0.283
5.我担心如果我没有保持忙碌状态, 会显得和大家格格不入 [I worry that if I don't stay busy, I will appear out of tune with others.]	−0.019	**0.633**	0.116
6. 我担心如果我不忙起来, 我会落后于别人 [I fear that if I don't keep myself busy, I will be left behind.]	−0.167	**0.963**	−0.005
7. 我担心当我工作/学习不够努力, 人们会不愿意和我合作 [I'm afraid that if I don't make enough effort in work or study, people will be unwilling to cooperate with me.]	0.024	**0.658**	0.137
8. 当感觉身体处于放松状态时, 我会感到害怕 [It scares me when my body feels relaxed.]	0.039	**0.416**	**0.483**
9. 当四肢感到沉重时, 我觉得害怕 [It scares me when my limbs feel heavy.]	0.018	0.067	**0.585**
10. 专注于自己的呼吸时, 我会感到惊慌害怕 [It frightens me to focus on my breathing.]	0.055	−0.129	**0.736**
11. 当我感觉身体好像放慢了节奏, 就会担心身体可能出现了严重问题 [When my body feels as if it has been slowed down, I worry that there might be something terribly wrong with me.]	−0.003	0.040	**0.760**
12. 放松时的那种轻飘飘的感觉让我害怕 [It scares me when I am relaxing and I feel like I'm floating.]	−0.089	0.091	**0.772**
13. 我害怕做放松活动, 因为这会让我感觉脆弱 [I'm scared of doing relaxing activities because they make me feel vulnerable.]	−0.035	−0.047	**0.944**
14. 当我尝试放松身体时, 我感到自己在逐渐失控 [When I try to relax my body, I feel like I'm losing control.]	−0.013	−0.031	**0.936**

*Factor loadings above 0.4 were bolded*.

**Figure 2:**
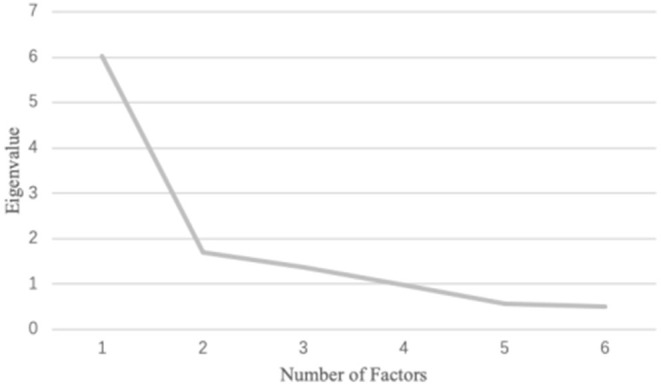
Screen plot for the second exploratory factor analysis with 13 items.

After removing item 8, the second EFA was conducted on the remaining 13 items. Similarly, the scree plot (Figure 2) showed clear curve at the 2-factor model; and 1-factor model had relatively low model fit (CFI = 0.846, TLI = 0.815, SRMR = 0.164, RMSEA = 0.173 [90% CI = 0.162–0.185]), and the 3-factor model (CFI = 0.970, TLI = 0.944, SRMR = 0.064, RMSEA = 0.095 [90% CI = 0.080–0.111]) fitted better than 2-factor model (CFI = 0.920, TLI = 0.882, SRMR = 0.108, RMSEA = 0.138 [90% CI = 0.125–0.152], see Table 3). Factors in the 3-factor model of the second EFA were consistent with those in the first EFA of this study. All the items met the cutoff of factor loadings, and no items were cross-loaded (see Table 4). Factor 1 (Items 1, 2, 3, and 4) was labeled as *Social appealing concerns* because the items were all related to concerns about other people's reactions to one's appearance when relaxing (e.g., appearing unattractive). The second factor contained three items (items 5, 6, and 7) related to ones' performance as evaluated by others (e.g., appearing out of tune with others) during relaxation and was named *Social performance concerns*. Factor 3 (Items 9, 10, 11, 12, 13, and 14) was called *Physical concerns*, representing mostly about “physical or bodily processes that may occur during relaxation,” with two items about cognitive concerns as labeled in the previous study (7).

**Table 3:** Model fit for EFA (13 items).

	***χ^2^***	***df***	**CFI**	**TLI**	**SRMR**	**RMSEA (90%CI)**
1-factor	674.416	65	0.846	0.815	0.164	0.173 [0.162–0.185]
2-factor	369.794	53	0.920	0.882	0.108	0.138 [0.125–0.152]
3-factor	160.509	42	0.970	0.944	0.064	0.095 [0.080–0.111]
4-factor	54.253	32	0.994	0.986	0.027	0.047 [0.024–0.068]
5-factor	18.59	23	1.000	1.004	0.016	0.000 [0.000–0.035]
6-factor	10.242	15	1.000	1.006	0.012	0.000 [0.000–0.035]

**Table 4:** Factor loadings for the second three-factor model.

**Item**	**Factor**		
	**F1**	**F2**	**F3**
1. 当我让身体放松时, 我担心我会失去外表吸引力 [I worry that when I let my body relax, I will look unattractive.]	**0.944**	0.008	−0.030
2. 如果我的身体放松下来, 我担心失去社交魅力/吸引力 [I fear that if my body is relaxed, I will not be socially appealing.]	**0.922**	−0.058	0.001
3. 我担心当我让身体放松时, 人们会取笑我 [I worry that when I let my body relax, people will make fun of me.]	**0.645**	−0.008	0.185
4. 我不喜欢放松, 因为这让我感到与他人失去了联系 [I do not like to relax because it makes me feel out of contact with others.]	**0.536**	0.091	0.251
5. 我担心如果我没有保持忙碌状态, 会显得和大家格格不入 [I worry that if I don't stay busy, I will appear out of tune with others.]	0.095	**0.637**	0.000
6. 我担心如果我不忙起来, 我会落后于别人 [I fear that if I don't keep myself busy, I will be left behind.]	−0.003	**0.976**	−0.167
7. 我担心当我工作/学习不够努力, 人们会不愿意和我合作 [I'm afraid that if I don't make enough effort in work or study, people will be unwilling to cooperate with me.]	0.133	**0.672**	0.031
9. 当四肢感到沉重时, 我觉得害怕 [It scares me when my limbs feel heavy.]	0.035	0.048	**0.588**
10. 专注于自己的呼吸时, 我会感到惊慌害怕 [It frightens me to focus on my breathing.]	0.040	−0.154	**0.777**
11. 当我感觉身体好像放慢了节奏, 就会担心身体可能出现了严重问题 [When my body feels as if it has been slowed down, I worry that there might be something terribly wrong with me.]	0.008	0.018	**0.770**
12. 放松时的那种轻飘飘的感觉让我害怕 [It scares me when I am relaxing and I feel like I'm floating.]	−0.043	0.021	**0.763**
13. 我害怕做放松活动, 因为这会让我感觉脆弱 [I'm scared of doing relaxing activities because they make me feel vulnerable.]	−0.047	−0.02 2	**0.939**
14. 当我尝试放松身体时, 我感到自己在逐渐失控 [When I try to relax my body, I feel like I'm losing control.]	−0.024	0.006	**0.928**

*F1, Social appealing concerns; F2, Social performance concerns; F3, Physical concerns. Factor loadings above 0.4 were bolded*.

#### Confirmatory Factor Analyses

The CFA on Sample 2 suggested moderately good model fit for the final three-factor model with 13 items (χ^2^ = 197.797, *df* = 62, χ^2^/df = 3.19, CFI = 0.963, TLI = 0.954, SRMR = 0.059, RMSEA = 0.088 [90% CI = 0.074–0.102]). Standardized factor loadings ranged from 0.615 to 0.940 (*M* = 0.795). The results by multi-group CFA demonstrated measure and structure invariance of the scale across gender, relaxation experience and age (see Supplementary Material B).

#### Internal Consistency Reliability

The internal consistency reliability was moderately good for the total scale (α = 0.857), *Social appealing concerns* (α = 0.848), *Social performance concerns* (α = 0.756) and *Physical concerns* (α = 0.796); the average inter-item correlations were generally moderate for all the sub-scales (*r*s = 0.409–0.584).

## Study 2

The purpose of this study was to test the test-retest reliability, convergent validity, and predictive validity of RSI. Referring to the previous study (7), we hypothesized that the RSI would demonstrate (1) adequate test-retest reliability and temporal stability; (2) good convergent validity in terms of positive correlations with anxiety symptoms, PTSD, negative affect, worry, and depression, and negative correlations with positive affect; and (3) good predictive criterion validity of sub-scales by uniquely predicting RIA and social anxiety.

### Method

#### Participants and Procedure

A subset of participants in Study 1 (*N* = 465, 37.6% males, *M*_age_ = 20.15, *SD* = 1.908) responded to the questionnaires in Study 2 2 weeks later.

#### Measures

The 13-item RSI, as developed in Study 1, was used in this study. The Cronbach's α for the RSI-total scale was 0.91 and for the three RSI sub-scales ranged from 0.84 to 0.88.

##### Anxiety Sensitivity Index-3

The ASI-3 (17) is an 18-item instrument used to assess the degree to which participants fear negative consequences caused by anxiety symptoms (example item: “It's important for me not to be nervous”). ASI-3 contains three dimensions: physical, psychological, and social concerns. Respondents rated their level of fear of arousal-related feelings on a 5-point scale (0 = very little to 4 = very much). Internal consistency for the total scale and three sub-scales in the current sample was good to excellent (αs = 0.83–0.93).

##### Interaction Anxiety

The 15-item instrument is used to assess the tendency to experience subjective social anxiety outside of behavior [example item: “Meetings often make me feel anxious and uncomfortable”; (18)]. Responses were rated on a 5-point scale (1 = not at all to 5 = extremely). Internal consistency for the total score in the current sample was .863.

##### Symptom Check-List 90 (SCL−90)

Anxiety, somatization, depression, and phobic anxiety symptoms scales from SCL-90 were used to assess convergent validity (example item: “Headache”), which is a Likert scale with anchors 0 = not at all to 5 = extremely (19). Cronbach's αs for these sub-scales in the current sample were good (ranging from 0.86 to 0.93).

##### The PTSD Checklist-Civilian Version

This scale is used to measure an individual's reaction and the severity of that reaction after experiencing or witnessing an unpredictable and unexpected event [example item: “Often have nightmares about this matter”; (20)]. Responses were rated on a 5-point scale (1 = not at all to 5 = extremely). Internal consistency for the total score in the current sample was 0.94.

##### Positive and Negative Affect Scale (PANAS)

The PANAS measures past week's positive [e.g., “Enthusiastic”] and negative affect [e.g., “Scared”; (21)]. Responses were rated on a 5-point scale (1 = not at all to 5 = extremely). Cronbach's alpha in the current sample was 0.873 for positive affect and 0.891 for negative affect.

##### Penn State Worry Questionnaire

The 16-item measure assesses the tendency to worry [example item: “Many situations make me worry”; (22)]. Responses were rated on a 5-point scale (1 = not at all to 5 = extremely). Internal consistency for the total score in the current sample was 0.896.

##### Relaxation-Induced Anxiety

To assess predictive criterion validity, we asked participants, “Do you ever feel increased anxiety when doing relaxing activities like mediation?.” This item was based on the item published in Luberto et al. (7). The responses were rated on a 4-point scale (1 = never or rarely to 4 = almost always) in the current sample.

##### Relaxation Experience

Participants were asked, “Do you have relaxation experiences such as mindfulness practice, yoga, etc.?” Responses were rated on “Yes” (1) or “No” (0).

Average scores were computed for the total scale of the above measures and the sub-scales of the RSI, ASI, and PANAS.

#### Analytic Approach

First, descriptive statistics were run for RSI total scale and all sub-scales. Second, test-retest reliability was assessed by exploring zero-order correlations between participants' RSI scores from the first and second study, and temporal stability was also examined by paired-samples *t*-tests. Third, we calculated zero-order correlation coefficients of RSI total score and subscale scores with all convergent validity measures. Finally, linear regression was used to examine the criterion validity of the RSI in terms of associations with RIA and social anxiety.

### Results

#### Descriptive Statistics

Table 5 demonstrated that, women scored higher than men on the *Social performance concerns* factor (*t* = −2.050, *p* = 0.041). There were no significant differences in the RSI total and sub-scales scores between students with or without relaxation experience (see Table 5). All RSI sub-scales scores were correlated with one another (range: 0.456 to 0.605, *p*s <0.001), as well as with the RSI total score (range: 0.783 to 0.867, *p*s < 0.001)(see Table 6).

**Table 5:** Descriptive statistics for RSI scores and their differences by sex and relaxation experience.

**RSI factor**	**Total sample**	**Sex**				**Relaxation experience**			
	***M (SD)***	**Men (0)**	**Women (1)**	***t***	**Cohen's** ***d***	**No (0)**	**Yes (1)**	***t***	**Cohen's** ***d***
		***M (SD)***	***M (SD)***			***M (SD)***	***M (SD)***		
1. RSI-SA	0.758	0.806	0.729	0.966	0.096	0.682	0.735	−0.752	0.071
	(0.804)	(0.859)	(0.769)			(0.740)	(0.750)		
2. RSI-SP	1.610	1.488	1.684	−2.050^*^	0.202	1.614	1.676	−0.749	0.070
	(0.974)	(1.044)	(0.924)			(0.918)	(0.848)		
3. RSI-PC	0.557	0.522	0.578	−0.860	0.083	0.481	0.475	0.126	0.011
	(0.665)	(0.637)	(0.681)			(0.555)	(0.507)		
4. RSI-total	0.862	0.833	0.878	−0.752	0.069	0.804	0.832	−0.568	0.053
	(0.653)	(0.672)	(0.642)			(0.546)	(0.500)		

* *p < 0.05. SA, Social appealing concerns; SP, Social performance concerns; PC, Physical concerns; Relaxation experience: “Do you have relaxation experiences such as mindfulness practice, yoga, etc.?”*

**Table 6:** Zero-order correlations among the RSI scores and measures of validity.

	**RSI-total**	**RSI-SA**	**RSI-SP**	**RSI-PC**
RSI-SA	0.867^**^	1	0.591^**^	0.605^**^
RSI-SP	0.783^**^	0.591^**^	1	0.456^**^
RSI-PC	0.856^**^	0.605^**^	0.456^**^	1
ASI-total	0.606^**^	0.471^**^	0.513^**^	0.535^**^
ASI-PC	0.520^**^	0.377^**^	0.412^**^	0.500^**^
ASI-CC	0.588^**^	0.447^**^	0.511^**^	0.517^**^
ASI-SC	0.545^**^	0.457^**^	0.475^**^	0.444^**^
Social anxiety	0.319^**^	0.245^**^	0.312^**^	0.252^**^
Anxiety	0.536^**^	0.400^**^	0.445^**^	0.493^**^
Phobic anxiety	0.474^**^	0.413^**^	0.333^**^	0.433^**^
Somatization	0.414^**^	0.289^**^	0.300^**^	0.428^**^
PTSD	0.432^**^	0.327^**^	0.336^**^	0.409^**^
Negative affect	0.566^**^	0.475^**^	0.432^**^	0.505^**^
Worry	0.520^**^	0.437^**^	0.491^**^	0.394^**^
Depression	0.590^**^	0.466^**^	0.468^**^	0.537^**^
Positive affect	−0.011	−0.003	0.007	−0.026

** *p < 0.01. SA, Social appealing concerns; SP, Social performance concerns; PC, Physical concerns; CC, Cognitive concerns; SC, Social concerns*.

#### Test-Retest Reliability

The test-retest reliability results of RSI total score and *Social performance concerns* were acceptable (*r* = 0.685 and 0.627 respectively, *p*s < 0.001), while *Social appealing concerns* and *Physical concerns* had low test-retest reliability (*r* = 0.525 and 0.557 respectively, *p*s < 0.001). In terms of temporal stability, we found no significant changes in scores for the *Social appealing concerns* [*M* = 0.703 at Time 1 and 0.758 at Time 2, *t* (464) = – 1.580, *p* = 0.115] or *Social performance concerns* sub-scales [*M* = 1.638 at Time 1 and 1.610 at Time 2, *t* (464) = −0.745, *p* = 0.457]. However, the scores on the total scale [*M* = 0.815 at Time 1 and 862 at Time 2, *t* (464) = −2.079, *p* = 0.038] and *Physical concerns* sub-scale [*M* = 0.479 at Time 1 and 0.557 at Time 2, *t* (464) = −2.911, *p* = 0.04] significantly increased at Time 2.

#### Convergent Validity

As shown in Table 6, the RSI total and sub-scales scores were positively correlated with the ASI-3 total and sub-scales scores. They were also positively correlated with social anxiety symptoms, anxiety symptoms, phobic anxiety symptoms, somatization symptoms, PTSD symptoms, negative affect, worry, and depression. Contrary to prediction, positive affect was not correlated with any of the RSI total or sub-scales scores (*ps* > 0.05).

#### Predictive Criterion Validity

Linear regressions demonstrated that, after controlling the effects of sex and negative affect, the RSI total scale (β = 0.178, *t* = 3.736, *p* < 0.001), *Physical concerns* (β = 0.215, *t* = 4.718, *p* < 0.001) and *Social appealing concerns* sub-scales (β = 0.123, *t* = 2.687, *p* < 0.05) predicted relaxation-induced anxiety, while the *Social performance concerns* sub-scale did not (β = 0.025, *t* = 0.526, *p* > 0.05) (Table 7). When anxiety sensitivity was included in the models, similarly, all RSI scores except for the *Social performance concerns* sub-scale score remained significant predictors; anxiety sensitivity did not significantly predict relaxation-induced anxiety in any of the models. After controlling the effects of sex and negative affect, *Social performance concerns* significantly predicted social anxiety, while *Social appealing concerns* and *Physical concerns* did not (Table 8).

**Table 7:** RSI and ASI-3 scores predicting relaxation-induced anxiety.

		**β**	***t***	***p***
Model 1	RSI-total	0.175	3.042	0.002
	ASI-total	0.005	0.087	0.930
Model 2	RSI-SA	0.108	2.189	0.029
	RSI-SP	−0.039	−0.769	0.442
	ASI-SC	0.083	1.611	0.108
Model 3	RSI-PC	0.237	4.613	<0.001
	ASI-PC	−0.050	−0.936	0.350

*SA, Social appealing concerns; SP, Social performance concerns; PC, Physical concerns; SC, Social concerns*.

**Table 8:** RSI scores predicting social anxiety.

	**β**	***t***	***p***
RSI-SA	0.019	0.303	0.762
RSI-SP	0.181	3.275	0.001
RSI-PC	0.057	1.008	0.314

*SA, Social appealing concerns; SP, Social performance concerns; PC, Physical concerns*.

## Discussion

The current study is the first study examining the factor structure of the RSI among the Chinese sample. In this study, three factors (i.e., *Social appealing concerns, Social performance concerns*, and *Physical concerns*) were identified following the EFA, and subsequent CFA revealed that the 3-factor structure had an overall good model fit. Measure and structure invariance as found by multi-group CFA also indicated the stability of the scale across gender, relaxation experience and age. The Chinese version RSI had good internal consistency reliability but relatively low 2-week test-retest reliability. Notably, females scored higher in *Social performance concerns* as newly extracted in the current study. Distinct from anxiety sensitivity, *Physical concerns*, and *Social appealing concerns* predicted relaxation-induced anxiety, while *Social performance concerns* solely predicted social anxiety. All RSI scores were correlated with most of the psychological symptoms.

Three factors (i.e., *Social appealing concerns, Social performance concerns*, and *Physical concerns*) were extracted based on the Chinese sample. Compared to the original version by Luberto et al. (7), *Physical concerns* remained the stable factor in the structure of relaxation sensitivity for the Chinese population. Meanwhile, some differences in the factor structure emerged in the current study. Specifically, items about *Social appealing concerns* constituted as a sub-factor of *Social concerns*. *The Social performance concerns* factor was additionally extracted, which was another aspect of *Social concern*s suggested by Luberto et al. (7). Separating the factor of *Social concerns* into these two factors would generate a clearer factor structure. *Social performance concerns*, which had higher average score than the other factors (*t* = −14.544, *p* < 0.001, Cohen's *d* = 0.954; *t* = −19.251, *p* < 0.001, Cohen's *d* = 1.263), would be a typical factor for the Chinese population. This finding may be related to the competition pressure cultivated in the Asian culture, with constant social comparison and fierce competition in daily life (11). The *Cognitive concerns* factor was not retained, which may be because mind and body are considered the same object (i.e., “body-mind-connection”) in Chinese culture. Specifically, the ancient Chinese emphasizes the mutual influence between mind and body and considers the body an essential role (23). This is also consistent with the result that two items from the *Cognitive concerns* sub-scale in Luberto et al. (7)'s study retained on the *Physical concerns* sub-scale of the Chinese version (i.e., “I'm scared of doing relaxing activities because they make me feel vulnerable,” and “When I try to relax my body, I feel like I'm losing control”).

All subfactors had moderate correlations with one another as well as high correlations with the RSI total score. This result suggests a latent structure of relaxation sensitivity involving related but distinct fears of physical and social consequences. CFA revealed that the 3-factor structure had a good model fit except for relatively high RMSEA. Although the high RMSEA index may be due to the highly asymmetric categorical distributions and small sample sizes (24), it may suggest that the factor structure for the Chinese version of RSI needs to be validated in the future. Especially, *Social performance concerns* had relatively lower correlations with the other two factors, which should be further addressed or verified. In sum, the *Physical concerns* factor included mostly physical concerns and some cognitive concerns items; hence, this factor is perhaps tapping into concerns about internal relaxation-related consequences. The different domains of social concerns are separated as different aspects of external concerns. Since this is the first independent validation of the measure, it may indicate a cultural difference or evidence for a different factor structure of RSI. Therefore, both the western and Chinese versions could be further explored for factor structures that reflect internal and external concerns as the higher-order factors of RSI.

Similar to the previous study by Luberto et al. (7), internal consistency reliability for the total scale and sub-scales ranged from moderate to moderately high. However, the 2-week test-retest reliability was moderately low and relatively lower than those in the previous study as measured within one week (7). Scores on the total scale and *Physical concerns* sub-scale significantly increased at Time 2, probably because college students had more pressure at Time 2 before the mid-term exams. The previous study by Luberto et al. (7) found a significant decrease in *Physical concerns* a week later. To sum up, these results may indicate that the *Physical concerns* may be prone to contextual influences. Especially, *Physical concerns* may have more weekly fluctuation than the other factors. As suggested by Luberto et al. (7), *Physical concerns* would depend on multiple contextual factors (e.g., physical status), which fluctuate over time and further influence emotional state (25). Besides, females had significantly higher *Social performance concerns* than males, probably because women were more easily affected by the feedback they received for self-evaluation than men, and thus were more sensitive to social comparison (26). No differences were found by sex or whether the participants had experience in relaxation-related practice (e.g., mindfulness practices, yoga). This result indicates that the RSI generally remains stable across sex and populations with or without experience of relaxation-related practice.

Moderate associations were found between RSI and ASI scales, with relatively higher associations between the corresponding sub-scales (i.e., ASI-PC and RSI-PC; ASI-SC and RSI-SP, SA). Consistent with the previous study by Luberto et al. (7), the unique variance of RSI (63~86%) was greater than the shared variance with anxiety sensitivity; relaxation sensitivity, but not anxiety sensitivity, had a significant prediction for relaxation-induced anxiety. These results further support Luberto 2021's theory that anxiety sensitivity and relaxation sensitivity are related but distinct constructs. Besides, the *Social performance concerns* did not significantly predict RIA, while solely predicted social anxiety. This finding may reflect that *Social performance concerns* is a unique factor mainly related to social anxiety, as also suggested by Hoffman (27) and Voncken et al. (28), and its relationship with RIA may be indirect or weaker. In the Contrast Avoidance Model, individuals with anxiety may fear the sharp increase of negative emotion and would rather keep feeling anxious during the process of relaxation (29). The current finding may imply that attention should also be paid to the individuals with the fear objects opposite to tension (i.e., relaxation). Exploring more theoretical explanations in this field could help individuals with different aspects of RIA benefit from relaxation training or mindfulness practice.

The current study found moderate associations of RSI with higher negative emotion, worry, and psychological symptoms (i.e., anxiety, somatization, PTSD, depression). Based on cognitive behavior theory, it may be that a higher level of relaxation sensitivity reduces the use of effective stress-reducing behaviors and mental flexibility, thereby increasing psychological problem symptoms (7, 30). In addition, individuals with PTSD are often in a state of hyperarousal (31), which may increase their difficulty in relaxing. Moreover, individuals with higher relaxation sensitivity may fear more about their somatic experience and then have negative inferential styles about self, which is the risk factor for depression (32, 33). In sum, the positive correlations with negative psychological outcomes indicated good convergent validity of RSI. Furthermore, across the three factors of RSI, *Physical concerns* had relatively higher correlations with the adverse outcomes, except that *Social performance concerns* had the highest correlations with social anxiety and worry. This result may indicate that most of the psychological symptoms have the strongest relations with individual's fear in their somatic experience (34), while social anxiety and worry are mostly related to the fear of social performance, which is consistent with the link between social anxiety and social performance (28).

Notably, neither the sub-scales nor the total scale of RSI was related to positive affect, which was inconsistent with the previous study based on a non-clinical sample (7). The bivariate models for affect may explain this: Positive experience is primarily related to positive aspects of well-being, whereas negative experiences are primarily related to negative aspects (35, 36). Further, East Asians may be attuned to broader and more negative consequences of a positive event due to their dialectical thinking style and thus consider these two types of emotions independent, especially in a pleasant situation (37). This explanation is also consistent with the nonsignificant correlation between positive and negative emotions as found in the current study (*r* = −0.403, *p* = 0.350).

Based on the original framework of relaxation sensitivity, the current study further adapts the RSI to the Chinese population. The current findings indicate that the fear of relaxation in Chinese culture has similarities and differences compared with the western population in factor structure, which strengthens the role of *Physical concerns*, and expands social concerns with the perspective of *Social performance concerns*. Replicating the findings in Luberto et al.'s study (7), the present study further demonstrates the associations of relaxation sensitivity with psychological symptoms. This result may add further information about the exact object of an individual's fear related to the adverse outcomes during relaxation, which is essential in tailoring the intervention programs to individuals with relaxation-related concerns (7).

Strengths notwithstanding, several limitations should be considered in future research. First, caution should be paid when applying the current version of RSI to other populations because the current sample was limited to Chinese college students. Secondly, in the future study for the clinical population, a broader factor structure, additionally including *Cognitive concerns* and higher-order structure among these factors, should be examined by high-order CFA. Specifically, fear of internal consequences may include physical and cognitive concerns, and fear of external consequences may include social performance and appealing concerns. Besides, factors influencing relaxation sensitivity could be further investigated based on different clinical groups or intervention groups, reflecting different RIA and social anxiety (1, 10, 27). Thirdly, considering the fluctuation of RSI scores, we need to pay attention to the assessment timing when comparing relaxation sensitivity as indicated by RSI. Especially, using EMA or taking multiple repeated assessments over time would be most informative for understanding the time frame of change in RSI scores. Measurement invariance (e.g., scalar and configural invariance) could be further investigated across populations in different cultures or with and without clinical symptoms.

## Data Availability Statement

The original contributions presented in the study are included in the article/Supplementary Material, further inquiries can be directed to the corresponding author.

## Ethics Statement

The studies involving human participants were reviewed and approved by Shenzhen University. The patients/participants provided their written informed consent to participate in this study.

## Author Contributions

JZ wrote the manuscript. CL revised the manuscript. QH and JK collected and analyzed data. LZ planned the study, provided guidance in the study design, data collection and data analysis, and revised the manuscript. JZ and AY revised the manuscript and provided a theoretical explanation of the results. All authors contributed to the article and approved the submitted version.

## Funding

This work was supported by the National Natural Science Foundation of China [grant numbers 31700982], the Guangdong 13th Five-Year Plan Foundation for Young Scholar in Philosophy and Social Science [grant number GD17YXL01] to JZ, and the Start-up Research Grant of Shenzhen University (20200807163056003), Start-up Research grant (8940206/0187), Start-Up Research Grant (Peacock Plan: 20191105534C) to LZ.

## Conflict of Interest

The authors declare that the research was conducted in the absence of any commercial or financial relationships that could be construed as a potential conflict of interest.

## Publisher's Note

All claims expressed in this article are solely those of the authors and do not necessarily represent those of their affiliated organizations, or those of the publisher, the editors and the reviewers. Any product that may be evaluated in this article, or claim that may be made by its manufacturer, is not guaranteed or endorsed by the publisher.
