# Supplementary material for: Validation of the Chinese Version of Relaxation Sensitivity Index: A Tool for Predicting Treatment Effect in Mindfulness Interventions
Source: Front Public Health. 2021 Dec 20;9:809572. doi: 10.3389/fpubh.2021.809572 (PMC8720785; doi:10.3389/fpubh.2021.809572)
Supplement: Supplementary file 1 [file Data_Sheet_1.docx]

Supplementary Material

# Supplementary Figures and Tables

## Supplementary Figures

**Supplementary Figure A1.** Scree plot for the first exploratory factor analysis with 26-item version

## Supplementary Tables

Table A1 Model fit for EFA (26 items)

|  | *χ^2^* | *df* | CFI | TLI | SRMR | RMSEA (90%CI) |
| --- | --- | --- | --- | --- | --- | --- |
| 1-factor | 786.087 | 299 | .936 | .931 | .096 | .072 (.066–.078) |
| 2-factor | 549.868 | 274 | .964 | .957 | .073 | .057 (.050–.064) |
| 3-factor | 381.885 | 250 | .983 | .978 | .057 | .041 (.033–.049) |
| 4-factor | 293.037 | 227 | .991 | .988 | .046 | .030 (.019–.040) |
| 5-factor | 258.014 | 205 | .993 | .989 | .041 | .029 (.016–.039) |
| 6-factor | 214.832 | 184 | .996 | .993 | .036 | .023 (.000–.035) |

Table A2 Factor loadings for the 2 and 3-factor model (26 items)

| Item | 2-factor model | | 3-factor model | | |
| --- | --- | --- | --- | --- | --- |
|  | F1 | F2 | F1 | F2 | F3 |
| 1. 当我让身体放松时，我担心我会失去外表吸引力。  [I worry that when I let my body relax, I will look unattractive.] | .008 | **.785** | .142 | **.732** | .110 |
| 2. 如果我的身体放松下来，我担心失去社交魅力/吸引力。  [I fear that if my body is relaxed, I will not be socially appealing.] | -.096 | **.838** | -.007 | **.739** | .231 |
| ^a^ 3. 当身体放松时，我会担心自己看起来很傻。  [I worry that when I let my body relax, I will look silly.] | **.319** | **.442** | **.413** | **.440** | .014 |
| 4.我担心当我让身体放松时，人们会取笑我。  [I worry that when I let my body relax, people will make fun of me.] | .360 | **.576** | **.444** | **.493** | .132 |
| 5. 我不喜欢放松，因为这让我感到与他人失去了联系。  [I do not like to relax because it makes me feel out of contact with others.] | .209 | **.551** | .193 | .268 | **.427** |
| ^a^ 6. 我害怕在别人面前放松，这样会让我显得很奇怪。  [It scares me when I am relaxing in public. Because it will make me look very weird.] | **.420** | **.422** | **.431** | .224 | **.277** |
| 7. 我担心如果我没有保持忙碌状态，会显得和大家格格不入。  [I worry that if I don’t stay busy, I will appear out of tune with others.] | .323 | **.587** | .320 | .115 | **.556** |
| ^a^ 8. 我担心如果我处于放松状态，会受到别人的排挤（别人会瞧不起我）。  [I worry that if I stay relaxed, I will be ostracized by others.] | **.502** | **.462** | **.508** | .215 | **.334** |
| 9. 我担心如果我不忙起来，我会落后于别人。  [I fear that if I don’t keep myself busy, I will be left behind.] | .016 | **.833** | .025 | -.005 | **.882** |
| 10. 我担心当我工作/学习不够努力，人们会不愿意和我合作。  [I’m afraid that if I don’t make enough effort in work or study, people will be unwilling to cooperate with me.] | -.023 | **.836** | -.001 | .024 | **.848** |
| 11. 当感觉身体处于放松状态时，我会感到害怕。  [It scares me when my body feels relaxed.] | **.764** | .125 | **.812** | .156 | -.058 |
| ^c^ 12. 当呼吸变得更深长缓慢时，我会感到害怕。  [It scares me when my breathing becomes deeper.] | **.803** | -.043 | **.832** | -.063 | -.05 |
| 13. 当四肢感到沉重时，我觉得害怕。  [It scares me when my limbs feel heavy.] | **.655** | -.075 | **.649** | -.221 | .073 |
| ^d^ 14我不喜欢冥想之类的活动，因为它们给我的身体带来的一些感觉。  [I do not like activities like meditation because of the way they make my body feel.] | **.426** | .184 | **.445** | .088 | .106 |
| 15. 专注于自己的呼吸时，我会感到惊慌害怕。  [It frightens me to focus on my breathing.] | **.844** | -.123 | **.856** | -.216 | .000 |
| 16. 当我感觉身体好像放慢了节奏，就会担心身体可能出现了严重问题。  [When my body feels as if it has been slowed down, I worry that there might be something terribly wrong with me.] | **.519** | .216 | **.501** | -.082 | **.311** |
| 17. 放松时的那种轻飘飘的感觉让我害怕。  [It scares me when I am relaxing and I feel like I’m floating.] | **.731** | .014 | **.751** | -.059 | .024 |
| ^e^ 18. 我害怕放松时声音显得比以前更大、更模糊、或更远。  [It frightens me when I’m relaxing and noises seem louder, muffled, or further away than they previously were.] | **.873** | -.120 | **.943** | .003 | -.244 |
| ^d^ 19. 我讨厌按摩，因为我不喜欢肌肉放松时所带来的感觉。  [I hate getting massages because of the feeling it creates when my muscles relax.] | **.413** | .156 | **.473** | **.274** | -.126 |
| ^e^ 20. 当放松时景象变得模糊，我担心自己出了问题。  [While I’m relaxing and images become fuzzy, I worry that something is wrong with me.] | **.829** | -.090 | **.824** | -.296 | .117 |
| ^f^ 21. 我不喜欢放松，因为它让我感到失去控制。  [I do not like to relax because it makes me feel out of control.] | **.603** | .294 | **.668** | .309 | -.013 |
| ^f^ 22. 我不喜欢放松，因为我不喜欢我思维变慢。  [I do not like to relax because I do not like when my thoughts slow down.] | **.601** | .259 | **.624** | .196 | .099 |
| 23. 我害怕做放松活动，因为这会让我感觉脆弱。  [I’m scared of doing relaxing activities because they make me feel vulnerable.] | **.861** | .081 | **.919** | .132 | -.106 |
| ^b^ 24. 我担心在我放松时，我会失去了对时间的感觉。  [It scares me when I am relaxing and begin to feel like I am losing a sense of time.] | .351 | .311 | .334 | -.006 | .361 |
| 25. 当我尝试放松身体时，我感到自己在逐渐失控。  [When I try to relax my body, I feel like I’m losing control.] | **.576** | .069 | **.609** | .117 | -.067 |
| ^f^ 26. 当专注于当下（而非过去或未来），我会感到紧张或焦虑。  [Focusing on the present moment rather than the future or the past makes me feel anxious.] | **.640** | .133 | **.637** | -.079 | .199 |

*Note*. In the 2-factor model: *F1*= Physical and Cognitive concerns; *F2* = Social concerns; In the 3-factor model: *F1* = Physical and Cognitive concerns; *F2* = Social appealing concerns; *F3* = Social performance concerns; *a,* deleted due to low loading; *b*, deleted due to cross loading; *c*, deleted due to lower loading than similar meaning item; *d*, deleted due to less prevalence among the Chinese; *e*, deleted due to difficult in understanding; *f*, deleted due to beyond the meaning expressed by physical concerns; Items 6-10 are developed in current study. Factor loadings above 0.4 were bolded.
