# Supplementary material for: Validation of the Chinese Version of Relaxation Sensitivity Index: A Tool for Predicting Treatment Effect in Mindfulness Interventions
Source: Front Public Health. 2021 Dec 20;9:809572. doi: 10.3389/fpubh.2021.809572 (PMC8720785; doi:10.3389/fpubh.2021.809572)
Supplement: Supplementary file 2 [file Data_Sheet_2.pdf]

Table B1

*Multi-Group CFA of the Chinese version of RSI with 13 Items across Gender*

| Model | Free Parameters | Comparison Model | $\Delta\chi^2$ | $\Delta df$ | $p$ | CFI | ΔCFI | TLI | RMSEA (90% CI) |
|---|---|---|---|---|---|---|---|---|---|
| M1: Configural invariance | – | – | – | – | – | – | – | – | – |
| M2: Metric invariance | – | – | – | – | – | – | – | – | – |
| M3: Scalar invariance | 90 | – | – | – | – | .964 | – | .967 | .085 [.072, .098] |
| M4: Structural invariance - Mean | 87 | M3 | 19.08 | 3 | < .001 | .961 | .003 | .965 | .088 [.075, .100] |
| M5: Structural invariance - (Co)variance | 84 | M3 | 1.04 | 6 | .984 | .965 | .001 | .969 | .082 [.070, .095] |
| **M6: Structural invariance - (Co)variance, Mean** | **81** | **M4** | **40.169** | **6** | **< .001** | **.970** | **.009** | **.974** | **.075 [.062, .088]** |
| | | **M5** | **22.129** | **3** | **< .001** | **.970** | **.005** | **.974** | **.075 [.062, .088]** |

*Note.* M1 and M2 could not be identified, indicating constraining configural and metric invariance (i.e., factor loadings) only did not have an acceptable model fit. The scalar invariance model (M3) that further constrained the thresholds equal across groups demonstrated an acceptable model fit. M4 and M5 had acceptable model fits and were further compared with those in M3. Model with equal means across the group (M4) did not deteriorate the model fit as compared to M3, ΔCFI < .010. Constraining (co)variance of the three latent variables to be equal across groups (M5) also did not deteriorate the model fit, ΔCFI < .010. We further tested M6 with both mean and co(variance) of latent variables constrained equal across groups and found M6 did not deteriorated the model fit as compared to M4 and M5, ΔCFIs < .010. Hence, we took M6 as the final model and concluded that the two groups demonstrated measure and structure invariance.

Final selected model bolded.

Table B2

*Standardized Factor Loadings for the Best-fitting Multi-Group CFA Model (M6) across Gender*

| | Standardized Factor Loadings | | | | | |
|---|---|---|---|---|---|---|
| | Males (*N* = 126) | | | Females (*N* = 163) | | |
| Items | F1 | F2 | F3 | F1 | F2 | F3 |
| 1. I worry that when I let my body relax, I will look unattractive. | 0.908 | – | – | 0.884 | – | – |
| 2. I fear that if my body is relaxed, I will not be socially appealing. | 0.939 | – | – | 0.928 | – | – |
| 3. I worry that when I let my body relax, people will make fun of me. | 0.789 | – | – | 0.825 | – | – |
| 4. I do not like to relax because it makes me feel out of contact with others. | 0.904 | – | – | 0.824 | – | – |
| 5. I worry that if I don't stay busy, I will appear out of tune with others. | – | 0.927 | – | – | 0.812 | – |
| 6. I fear that if I don't keep myself busy, I will be left behind. | – | 0.769 | – | – | 0.660 | – |
| 7. I'm afraid that if I don't make enough effort in work or study, people will be unwilling to cooperate with me. | – | 0.732 | – | – | 0.708 | – |
| 9. It scares me when my limbs feel heavy. | – | – | 0.753 | – | – | 0.685 |
| 10. It frightens me to focus on my breathing. | – | – | 0.656 | – | – | 0.582 |
| 11. When my body feels as if it has been slowed down, I worry that there might be something terribly wrong with me. | – | – | 0.762 | – | – | 0.760 |
| 12. It scares me when I am relaxing and I feel like I'm floating. | – | – | 0.898 | – | – | 0.777 |
| 13. I'm scared of doing relaxing activities because they make me feel vulnerable. | – | – | 0.953 | – | – | 0.865 |
| 14. When I try to relax my body, I feel like I'm losing control. | – | – | 0.922 | – | – | 0.757 |
| Latent Factors | | | | | | |
| Factor means | 0[a] | 0[a] | 0[a] | 0[a] | 0[a] | 0[a] |
| Factor variances | 1 | 1 | 1 | 1 | 1 | 1 |
| Factor correlation | 0.557 (f2) | 0.635 (f3) | 0.689 (f1) | 0.557 (f2) | 0.635 (f3) | 0.689 (f1) |

[a] fixed for model identification.

*Note.* All free parameters were significant at $p < .001$ level. *F1* = Social appealing concerns; *F2* = Social performance concerns; *F3* = Physical concerns.

Table B3

*Multi-Group CFA of the Chinese version of RSI with 13 Items Across Groups with and without Relaxation Experience*

| Model | Free Parameters | Comparison Model | $\Delta\chi^2$ | $\Delta df$ | $p$ | CFI | ΔCFI | TLI | RMSEA (90% CI) |
|---|---|---|---|---|---|---|---|---|---|
| M1: Configural invariance | – | – | – | – | – | – | – | – | – |
| M2: Metric invariance | – | – | – | – | – | – | – | – | – |
| M3: Scalar invariance | 90 | – | – | – | – | .975 | – | .977 | .070 [.056, .084] |
| M4: Structural invariance - Mean | 87 | M3 | 6.314 | 3 | .097 | .977 | .002 | .979 | .067 [.052, .080] |
| M5: Structural invariance - (Co)variance | 84 | M3 | 11.47 | 6 | .075 | .979 | .004 | .981 | .064 [.049, .077] |
| **M6: Structural invariance - (Co)variance, Mean** | **81** | **M4** | **21.02** | **6** | **< .01** | **.983** | **.006** | **.985** | **.057 [.042, .071]** |
| | | **M5** | **15.86** | **3** | **< .01** | **.983** | **.004** | **.985** | **.057 [.042, .071]** |

*Note.* M1 and M2 could not be identified, and M3 demonstrated an acceptable model fit. Neither M4 nor M5 deteriorated the model fit as compared to M3, ΔCFIs < .010. We further tested M6 (with both mean and co(variance) of latent variables constrained equal across groups) and found M6 did not deteriorated the model fit as compared to M4 and M5, ΔCFIs < .010. Hence, M6 was chosen as the final model, indicating that the two groups demonstrated measure and structure invariance.

Final selected model bolded.

Table B4

*Standardized Factor Loadings for the Best-fitting Multi-Group CFA Model (M6) across Groups with and without Relaxation Experience*

| | Standardized Factor Loadings | | | | | |
|---|---|---|---|---|---|---|
| | Without Relaxation Experience (*N* = 173) | | | With Relaxation Experience (*N* = 116) | | |
| Items | F1 | F2 | F3 | F1 | F2 | F3 |
| 1. I worry that when I let my body relax, I will look unattractive. | 0.925 | – | – | 0.854 | – | – |
| 2. I fear that if my body is relaxed, I will not be socially appealing. | 0.970 | – | – | 0.904 | – | – |
| 3. I worry that when I let my body relax, people will make fun of me. | 0.759 | – | – | 0.838 | – | – |
| 4. I do not like to relax because it makes me feel out of contact with others. | 0.828 | – | – | 0.884 | – | – |
| 5. I worry that if I don't stay busy, I will appear out of tune with others. | – | 0.836 | – | – | 0.816 | – |
| 6. I fear that if I don't keep myself busy, I will be left behind. | – | 0.730 | – | – | 0.741 | – |
| 7. I'm afraid that if I don't make enough effort in work or study, people will be unwilling to cooperate with me. | – | 0.753 | – | – | 0.732 | – |
| 9. It scares me when my limbs feel heavy. | – | – | 0.720 | – | – | 0.754 |
| 10. It frightens me to focus on my breathing. | – | – | 0.593 | – | – | 0.607 |
| 11. When my body feels as if it has been slowed down, I worry that there might be something terribly wrong with me. | – | – | 0.754 | – | – | 0.856 |
| 12. It scares me when I am relaxing and I feel like I'm floating. | – | – | 0.813 | – | – | 0.771 |
| 13. I'm scared of doing relaxing activities because they make me feel vulnerable. | – | – | 0.917 | – | – | 0.905 |
| 14. When I try to relax my body, I feel like I'm losing control. | – | – | 0.802 | – | – | 0.867 |
| Latent Factors | | | | | | |
| Factor means | 0[a] | 0[a] | 0[a] | 0[a] | 0[a] | 0[a] |
| Factor variances | 1 | 1 | 1 | 1 | 1 | 1 |
| Factor correlation | 0.523 (f2) | 0.604 (f3) | 0.670 (f1) | 0.523 (f2) | 0.604 (f3) | 0.670 (f1) |

[a] fixed for model identification.

*Note*. All free parameters were significant at $p < .001$ level. *F1* = Social appealing concerns; *F2* = Social performance concerns; *F3* = Physical concerns.

Table B5

*Multi-Group CFA of the Chinese version of RSI with 13 Items across Age Groups*

| Model | Free Parameters | Comparison Model | $\Delta\chi^2$ | $\Delta df$ | $p$ | CFI | ΔCFI | TLI | RMSEA (90% CI) |
|---|---|---|---|---|---|---|---|---|---|
| M1: Configural invariance | – | – | – | – | – | – | – | – | – |
| M2: Metric invariance | – | – | – | – | – | – | – | – | – |
| M3: Scalar invariance | 90 | – | – | – | – | .967 | – | .970 | .076 [.063, .089] |
| M4: Structural invariance - Mean | 87 | M3 | 9.393 | 3 | < .05 | .970 | .003 | .973 | .072 [.059, .086] |
| M5: Structural invariance - (Co)variance | 84 | M3 | 4.591 | 6 | .597 | .967 | .000 | .971 | .075 [.061, .088] |
| | | **M4** | **17.04** | **6** | **< .01** | **.967** | **.000** | **.971** | **.074 [.061, .088]** |
| **M6: Structural invariance - (Co)variance, Mean** | **81** | **M5** | **3.052** | **3** | **.384** | **.967** | **.000** | **.971** | **.074 [.061, .088]** |

*Note.* According to the data distribution, participants were classified into two age groups (the group with age > 20 coded as 1, else as 0). M1 and M2 could not be identified, and M3 demonstrated an acceptable model fit. M4 and M5 did not deteriorated the model fit as compared to M3, ΔCFIs < .010. M6 did not deteriorated the model fit as compared to M4 and M5, ΔCFIs < .010. Hence, M6 was chosen as the final model, indicating that the two groups demonstrated measure and structure invariance.
Final selected model bolded.

Table B6

*Standardized Factor Loadings for the Best-fitting Multi-Group CFA Model (M6) across Age Groups*

| | Standardized Factor Loadings | | | | | |
|---|---|---|---|---|---|---|
| | Age <= 20 (*N* = 200) | | | Age > 20 (*N* = 87) | | |
| Items | F1 | F2 | F3 | F1 | F2 | F3 |
| 1. I worry that when I let my body relax, I will look unattractive. | 0.880 | – | – | 0.937 | – | – |
| 2. I fear that if my body is relaxed, I will not be socially appealing. | 0.943 | – | – | 0.918 | – | – |
| 3. I worry that when I let my body relax, people will make fun of me. | 0.790 | – | – | 0.774 | – | – |
| 4. I do not like to relax because it makes me feel out of contact with others. | 0.836 | – | – | 0.875 | – | – |
| 5. I worry that if I don't stay busy, I will appear out of tune with others. | – | 0.819 | – | – | 0.880 | – |
| 6. I fear that if I don't keep myself busy, I will be left behind. | – | 0.743 | – | – | 0.732 | – |
| 7. I'm afraid that if I don't make enough effort in work or study, people will be unwilling to cooperate with me. | – | 0.738 | – | – | 0.740 | – |
| 9. It scares me when my limbs feel heavy. | – | – | 0.709 | – | – | 0.748 |
| 10. It frightens me to focus on my breathing. | – | – | 0.603 | – | – | 0.597 |
| 11. When my body feels as if it has been slowed down, I worry that there might be something terribly wrong with me. | – | – | 0.765 | – | – | 0.802 |
| 12. It scares me when I am relaxing and I feel like I'm floating. | – | – | 0.794 | – | – | 0.884 |
| 13. I'm scared of doing relaxing activities because they make me feel vulnerable. | – | – | 0.903 | – | – | 0.950 |
| 14. When I try to relax my body, I feel like I'm losing control. | – | – | 0.774 | – | – | 0.774 |
| Latent Factors | | | | | | |
| Factor means | 0[a] | 0[a] | 0[a] | 0[a] | 0[a] | 0[a] |
| Factor variances | 1 | 1 | 1 | 1 | 1 | 1 |
| Factor correlation | 0.526 (f2) | 0.617 (f3) | 0.661 (f1) | 0.526 (f2) | 0.617 (f3) | 0.661 (f1) |

[a] fixed for model identification.

*Note.* All free parameters were significant at $p < .001$ level. *F1* = Social appealing concerns; *F2* = Social performance concerns; *F3* = Physical concerns.
